# Sieved mass and shape data from simulated fluvial transport of icy clasts in the Titan Tumbler

**DOI:** 10.1016/j.dib.2022.107815

**Published:** 2022-01-11

**Authors:** Anthony D. Maue, Joseph S. Levy, Devon M. Burr, Patrick R. Matulka, Erica Nathan

**Affiliations:** a Department of Astronomy and Planetary Science, Northern Arizona University, 527 S. Beaver St., Flagstaff, AZ 86011, United States; b Department of Geology, Colgate University, 13 Oak Drive, Hamilton, NY 13346, United States; c Department of Earth and Planetary Sciences, Washington University in St. Louis, 1 Brookings Drive, St. Louis, MO 63130, United States; d Department of Earth, Environmental and Planetary Sciences, Brown University, Box 1846, 324 Brook Street, Providence, RI 02912, United States

**Keywords:** Geoscience, Planetary surface processes, Tumbling barrel, Titan, Ices, Fluvial abrasion, Downstream fining, Cryogenics

## Abstract

Data in this article are related to the research article “Rapid rounding of icy clasts during simulated fluvial transport in the Titan Tumbler”. Whereas that research focused on low-temperature ice abrasion in the context of Saturn's moon Titan, the full dataset on experiments testing the breakdown of water ice under a variety of tested conditions is reported in this article. Following the work of previous terrestrial studies, these experiments utilize tumblers that produce collisions to simulate some aspects of mechanical weathering during fluvial transport. Data files publicly available on Mendeley Data include measures of mass and roundness of clasts of specific grain sizes as well as raw images, videos, and the MATLAB script used for analysis. In this article, the varying conditions of temperature, initial clast size, shape, ice type, number of clasts for each of the 42 experiments are reported, along with best-fit models of abrasion typically applied in terrestrial tumbler studies. This text describes the methodology, including the development of icy clasts, operation of the tumblers, measurement of clast properties, calculation of derived parameters, and application of abrasion models. Exploration of various approaches to tumbler development and data acquisition are reported to benefit future researchers in this area. Experiments on the abrasion of different materials benefit from cross-comparison, which is also a fundamental aspect of planetary science.

## Specifications Table

**Table:** 

Subject	Space and planetary science
Specific subject area	Fluvial transport, icy sedimentary processes, planetary surface processes, cryogenic laboratory experiments, clast size and shape evolution
Type of data	Table, Image, Video
How data were acquired	Cubic and nonequant water-ice clasts were created through freezing of ice and through crushing with a ZENY Ice Shaver Crusher 300 W machine, sieving, and refreezing of ice grains. Abrasion experiments were performed in the dual 11-cm-diameter styrene-butadiene rubber (SBR) barrels of a Lortone 33B Rotary Tumbler and in the 15 cm-diameter polyvinyl chloride (PVC) barrel of a custom tumbler apparatus. Sediment in the tumblers was sieved and mass for each size fraction was measured on an Ohaus Scout Pro SP4001 scale. Images and video were taken with an iPhone camera and image analysis was done in MATLAB with a script from Manga et al. [1].
Data format	Raw and analyzed
Parameters for data collection	Data were collected in two different barrels regulated at three different temperatures. Icy clasts had initial variation in number, size, shape, and ice type. The size of sediment removed from the system at each measurement interval also varied across experiments.
Description of data collection	Clasts were sieved, weighed, and imaged before tumbling. After tumbling some distance, at each measurement interval, icy sediment was brushed out of the tumbler and into sieves. Each grain size bin was weighed and the largest bins (generally 4–8, 8–16, and >16 mm) were imaged. Clasts from the 16 mm sieve were imaged on three sides. Sediment coarser than a selected threshold size were returned to the barrel for further tumbling. Clast masses were used to estimate Sternberg- [2] and Mikoš- [3] style abrasion coefficients and images were analyzed in MATLAB to compute roundness indices.
Data source location	Institution: Colgate University City/Town/Region: Hamilton, New York Country: United States
Data accessibility	Repository name: Mendeley Data Data identification number: http://dx.doi.org/10.17632/hnnmw6ygf3 Direct link to the dataset: https://data.mendeley.com/datasets/hnnmw6ygf3
Related research article	A.D. Maue, J.S. Levy, D.M. Burr, P.R. Matulka, E. Nathan (2022). Rapid rounding of icy clasts during simulated fluvial transport in the Titan Tumbler, *Icarus* 375. 114831. https://doi.org/10.1016/j.icarus.2021.114831

## Value of the Data

• These data are useful because they provide the complete measurements of changes to icy sediment under a variety of conditions previously unexplored, in a format that can be readily analyzed.
• These data benefit researchers interested in the breakdown of sediment on Titan or how it might compare to that on other planetary bodies.
• These data can be used with analytical methods not applied in the Icarus research article [4] to quantify aspects such as the distance travelled, abrasion rate, and/or clast shape in different ways.
• Future experiments may benefit from the description of issues encountered in the development and use of this cryogenic version of a classic instrument in sedimentary geology.

## 1 Data Description

These data describe the evolution of icy clasts during tumbler experiments performed across a variety of tested parameters. The experimental conditions for each run are reported in Table 1 and in the **Supplementary Material**. Experiments were performed with three different tumbler configurations, at three different temperatures, with two different types of ice, and with various initial conditions for the tumbled sediment.

**Table 1:** Configurations tested. Reference codes follow AbbbCCddEEffX where A is barrel type (S for SBR, R for R-PVC, or P for PVC), bbb is temperature (253 K for -20 °C freezer, 195 K for dry ice, ∼100 K for liquid nitrogen), CC is ice type (ig for polycrystalline igneous, fc for fine sand to coarse sand at 0.125–1 mm, me for medium sand at 0.25–0.5 mm, co for coarse sand at 0.5–1 mm, vc for very coarse sand at 1 and 2 mm, sg for very coarse sand to very fine gravel at 1–4 mm, gr for very fine gravel at 2–4 mm), dd is number of initial clasts, EE is shape (eq for equant, ne for nonequant), ff is size (in mm, or va for variable), and X is a letter denoting the iteration of that configuration. Barrel compositions are abbreviated where SBR is styrene-butadiene rubber and R-PVC is rubberized PVC (polyvinyl chloride). *D*_barrel_ refers to the barrel diameter, *T* to the intended temperature, *N*_clast_ to the initial number of clasts, *x* to the estimated distance tumbled, *D*_clast_ to the initial diameter of the clasts, ice seed type to the size of ice grains that comprise the clasts, shape to the initial shape of the clasts, *D*_filter_ to the size of clasts that were removed at each measurement interval. Also reported in **Supplementary Material** with minimum and maximum initial clast sizes given in two separate columns. Modified from Maue et al. [4].

Ref. Code	Barrel	*D*_barrel_ (cm)	*T* (K)	*N*_clast_	*x* (km)	*D*_clast_ (cm)	Ice seed type (mm)	Shape	*D*_filter_ (mm)
S253ig02eq33A	SBR	11	253	2	4.8	3.3	mixed	equant	n/a
S253ig04eq33A	SBR	11	253	4	24.8	3.3	mixed	equant	n/a
S253ig05eq33A	SBR	11	253	5	39.2	3.3	mixed	equant	n/a
S253ig03eq33A	SBR	11	253	3	18.35	3.3	mixed	equant	n/a
S253vc03eq33A	SBR	11	253	3	18.35	3.3	1–2	equant	n/a
S253co05eq33A	SBR	11	253	5	23.09	3.3	0.5–1	equant	n/a
S253me05eq33A	SBR	11	253	5	23.09	3.3	0.25–0.5	equant	n/a
S253ig05eq33B	SBR	11	253	5	31.1	3.3	mixed	equant	n/a
S253ig05eq33C	SBR	11	253	5	31.1	3.3	mixed	equant	n/a
P195vc01eq33A	PVC	15	195	1	1.84	3.3	1–2	equant	n/a
P195vc02eq33A	PVC	15	195	2	2.39	3.3	1–2	equant	n/a
P195vc06eq33A	PVC	15	195	6	16.73	3.3	1–2	equant	n/a
P195vc12eq33A	PVC	15	195	12	1.84	3.3	1–2	equant	n/a
R195vc01eq33A	R-PVC	15	195	1	0.92	3.3	1–2	equant	n/a
R195vc02eq33A	R-PVC	15	195	2	0.92	3.3	1–2	equant	n/a
R195vc06eq33A	R-PVC	15	195	6	0.92	3.3	1–2	equant	n/a
R195vc12eq33A	R-PVC	15	195	12	0.92	3.3	1–2	equant	n/a
P100ig06eq33A	PVC	15	100	6	0.81	3.3	mixed	equant	∼4
P100ig06eq33B	PVC	15	100	6	0.81	3.3	mixed	equant	∼4
P100ig07nevaA	PVC	15	100	7	1.08	∼2–8	mixed	noneq.	∼4
P100ig06eq33C	PVC	15	100	6	2.43	3.3	mixed	equant	∼4
P100ig06eq33D	PVC	15	100	6	4.32	3.3	mixed	equant	∼4
P100ig07nevaB	PVC	15	100	7	1.62	∼2–8	mixed	noneq.	∼4
P100ig04eq33A	PVC	15	100	4	1.78	3.3	mixed	equant	∼4
P100ig06eq33E	PVC	15	100	6	2.16	3.3	mixed	equant	∼4
P100gr06eq33A	PVC	15	100	6	1.89	3.3	2–4	equant	∼4
P100ig04eq45A	PVC	15	100	4	2.16	4.5	mixed	equant	∼4
P100ig04eq57A	PVC	15	100	4	1.89	5.7	mixed	equant	∼4
P100ig06eq33F	PVC	15	100	6	1.89	3.3	mixed	equant	16
P100ig06eq33G	PVC	15	100	6	1.35	3.3	mixed	equant	4
P100ig04eq57B	PVC	15	100	4	1.35	5.7	mixed	equant	2
P100ig04eq57C	PVC	15	100	4	2.75	5.7	mixed	equant	0
P100ig06eq33H	PVC	15	100	6	1.89	3.3	mixed	equant	1
P100ig06eq33I	PVC	15	100	6	1.89	3.3	mixed	equant	1
P100ig06eq33J	PVC	15	100	6	5.20	3.3	mixed	equant	1
P100ig03eq33A	n/a	n/a	100	3	0.00	3.3	mixed	equant	16
P100ig03eq33B	PVC	15	100	3	1.08	3.3	mixed	equant	1
P100sg06eq33A	PVC	15	100	6	4.05	3.3	1–4	equant	1
P100ig94nevaA	PVC	15	100	94	8.73	∼0.8–1.6	mixed	noneq.	1
P100ig55nevaA	PVC	15	100	55	7.56	∼0.8–7	mixed	noneq.	∼4
P100fc06eq33A	PVC	15	100	6	1.89	3.3	0.125–1	equant	∼4
P100fc06eq33B	PVC	15	100	6	1.62	3.3	0.125–1	equant	1

The measured roundness indices and mass for clasts at each step of a given run can be found in the **Supplementary Material**. At each cumulative timestep, the estimated total distance is reported based on the barrel's spin speed. Subsequent columns report the mass of each sieved grain size range and roundness statistics. Mass columns report that of grains with sizes between that of the given column header and the next filled column to the left. The smallest size bin (typically the pan) includes all sizes less than the next column to the left that has data, and the largest size bin reported includes all sizes larger than that of the given column header. Column headers are standardized between data files so sieve sizes that were not used for a given run are filled with values of -9999. In experiments where measurements were made in a walk-in freezer, the scale could be perturbed by high air flow and cold temperatures such that reported mass may have lower precision. For sieves that could be seen to contain sediment but did not register on the scale, “trace” is used. For sieves that did not contain sediment but registered mass, values were corrected to zero. Some reported mass values were negative after subtracting the known sieve mass from the mass of the sieve containing sediment and demonstrate an approximate error in these measurements up to ∼1 g.

For the coarsest sizes, statistics for the roundness indices are reported as mean, median, and standard deviation where available. These values apply to grain sizes larger than the largest available grain size and grain sizes between that of the given column header and the next left column header. The null value of -9999 was also used for size ranges of roundness indices that were not present or not imaged, and for the standard deviation of roundness indices for size ranges that included only a single clast.

Images used to extract roundness indices are shared in the **Supplementary Material**. Clasts >16 mm typically have images from three different perspectives. Clasts 4–8 and 8–16 mm, sizes for which images were collected in the coldest temperature experiments, are each imaged in one view.

Mass measurements are used to compute the fit of two exponential decay models for sediment comminution. The best-fit abrasion coefficients and associated goodness-of-fit parameters from the Sternberg [2] model and Mikoš [3] model are reported in Tables 2 and 3, respectively, as well as in the **Supplementary Material**. These fits were made in MATLAB using up to 100,000 iterations of the trust-region-reflective least squares algorithm. Experiments at 195 and 253 K (-78 and -20 °C) experienced no fragmentation (e.g., splitting) so model fits are only applied to the original input clasts (reported as >16 mm) as they gradually lose mass by attrition (e.g., grinding). Fragmentation into broad grain size distributions became more prominent during the experiments at ∼100 K (-170 °C), so a discrete grain size was selected for applying the mass-loss models. In early experiments at this temperature that involved no sieving, the models were applied to the mass loss of clasts greater than roughly 4 mm. In later experiments at ∼100 K that used sieving, the models were applied to the mass loss of both a defined fine and coarse grain size (usually >1 and >16 mm).

**Table 2:** Best-fit abrasion coefficient *k* from the Sternberg [2] model (Eq. (1)), root mean square error (RMSE), chi-squared, and reduced chi-squared as computed for the two grain size ranges, where applicable, for tumbler experiments. Two experiments are not included in this table; no mass measurements were recorded for P100ig06eq33A and the clasts in P100ig03eq33A were not tumbled. The values in this table are reported with greater precision in the **Supplementary Material** for this article.

Reference Code	Fine grain size (mm)	Coarse grain size (mm)	*k*_fine_ (km^−1^)	RMSE	χ2	χ2_ν_	*k*_coarse_ (km^−1^)	RMSE	χ2	χ2_ν_
S253ig02eq33A	n/a	16	n/a	n/a	n/a	n/a	5.2E−03	1.1E−03	8.8E−02	1.3E−02
S253ig04eq33A	n/a	16	n/a	n/a	n/a	n/a	1.3E−02	1.9E−02	2.6E+01	4.4E+00
S253ig05eq33A	n/a	16	n/a	n/a	n/a	n/a	1.2E−02	1.5E−02	2.8E+01	2.5E+00
S253ig03eq33A	n/a	16	n/a	n/a	n/a	n/a	4.7E−04	3.1E−03	7.5E−01	1.1E−01
S253vc03eq33A	n/a	16	n/a	n/a	n/a	n/a	1.1E−03	5.0E−03	2.0E+00	2.8E−01
S253co05eq33A	n/a	16	n/a	n/a	n/a	n/a	2.6E−03	2.2E−02	3.4E+01	5.7E+00
S253me05eq33A	n/a	16	n/a	n/a	n/a	n/a	2.3E−03	1.7E−02	2.1E+01	3.4E+00
S253ig05eq33B	n/a	16	n/a	n/a	n/a	n/a	2.1E−03	3.1E−02	4.0E+01	1.3E+01
S253ig05eq33C	n/a	16	n/a	n/a	n/a	n/a	5.0E−03	4.5E−02	8.2E+01	2.7E+01
P195vc01eq33A	n/a	16	n/a	n/a	n/a	n/a	6.4E−01	9.1E−02	6.6E+02	9.4E+01
P195vc02eq33A	n/a	16	n/a	n/a	n/a	n/a	6.7E−01	1.2E−01	1.7E+03	1.7E+02
P195vc06eq33A	n/a	16	n/a	n/a	n/a	n/a	2.4E−01	1.4E−01	2.5E+03	2.3E+02
P195vc12eq33A	n/a	16	n/a	n/a	n/a	n/a	4.1E−01	3.7E−02	1.1E+02	1.6E+01
R195vc01eq33A	n/a	16	n/a	n/a	n/a	n/a	7.3E−01	7.6E−02	2.3E+02	7.6E+01
R195vc02eq33A	n/a	16	n/a	n/a	n/a	n/a	4.7E−01	5.5E−02	1.2E+02	4.0E+01
R195vc06eq33A	n/a	16	n/a	n/a	n/a	n/a	3.7E−01	2.4E−02	2.3E+01	7.8E+00
R195vc12eq33A	n/a	16	n/a	n/a	n/a	n/a	3.7E−01	2.5E−02	2.5E+01	8.4E+00
P100ig06eq33B	4	n/a	2.2E−01	1.9E−02	1.1E+01	5.4E+00	n/a	n/a	n/a	n/a
P100ig07nevaA	4	n/a	2.1E−01	2.6E−02	2.8E+01	9.3E+00	n/a	n/a	n/a	n/a
P100ig06eq33C	4	n/a	1.4E−01	3.2E−02	9.2E+01	1.1E+01	n/a	n/a	n/a	n/a
P100ig06eq33D	4	n/a	8.2E−02	1.8E−02	4.9E+01	3.3E+00	n/a	n/a	n/a	n/a
P100ig07nevaB	4	n/a	1.6E−01	3.3E−02	6.6E+01	1.3E+01	n/a	n/a	n/a	n/a
P100ig04eq33A	4	n/a	1.4E−01	2.4E−02	4.0E+01	6.6E+00	n/a	n/a	n/a	n/a
P100ig06eq33E	4	n/a	1.4E−01	2.5E−02	4.9E+01	7.1E+00	n/a	n/a	n/a	n/a
P100gr06eq33A	4	n/a	1.2E−01	1.9E−02	2.6E+01	4.4E+00	n/a	n/a	n/a	n/a
P100ig04eq45A	4	n/a	2.6E−01	6.6E−02	3.4E+02	4.9E+01	n/a	n/a	n/a	n/a
P100ig04eq57A	4	n/a	4.0E−01	6.2E−02	2.7E+02	4.5E+01	n/a	n/a	n/a	n/a
P100ig06eq33F	1	16	1.7E−01	3.3E−02	7.7E+01	1.3E+01	6.3E−01	6.5E−02	3.0E+02	5.0E+01
P100ig06eq33G	1	16	2.0E−01	1.2E−02	7.5E+00	1.9E+00	1.6E−01	2.4E−02	3.0E+01	7.5E+00
P100ig04eq57B	1	16	3.2E−01	2.9E−02	4.1E+01	1.0E+01	4.7E−01	5.5E−02	1.5E+02	3.8E+01
P100ig04eq57C	1	16	2.6E−01	6.1E−02	2.6E+02	4.3E+01	3.6E−01	8.0E−02	4.5E+02	7.4E+01
P100ig06eq33H	1	16	1.3E−01	2.7E−02	5.2E+01	8.6E+00	3.4E−01	1.5E−02	1.7E+01	2.8E+00
P100ig06eq33I	1	16	1.1E−01	1.8E−02	2.3E+01	3.9E+00	1.2E−01	2.1E−02	3.0E+01	5.0E+00
P100ig06eq33J	1	16	6.5E−02	4.4E−02	1.4E+02	2.3E+01	9.0E−01	3.9E−02	1.1E+02	1.8E+01
P100ig03eq33B	1	16	1.5E−01	7.6E−03	2.3E+00	7.7E−01	8.2E−01	9.3E−02	3.5E+02	1.2E+02
P100sg06eq33A	1	16	8.0E−02	4.9E−02	1.7E+02	2.9E+01	6.1E−01	6.2E−02	2.7E+02	4.5E+01
P100ig94nevaA	1	8	3.7E−02	4.6E−02	3.8E+02	2.3E+01	2.4E−01	1.0E−01	1.8E+03	1.1E+02
P100ig55nevaA	1	16	7.5E−02	5.9E−02	2.8E+02	4.0E+01	8.8E−02	7.0E−02	3.9E+02	5.6E+01
P100fc06eq33A	1	16	1.0E−01	2.3E−02	3.8E+01	6.4E+00	1.1E−01	2.6E−02	4.9E+01	8.1E+00
P100fc06eq33B	1	16	3.9E−01	6.4E−02	2.5E+02	4.9E+01	5.4E−01	6.5E−02	2.5E+02	5.0E+01

**Table 3:** Best-fit coefficients from the Mikoš [3] model (Eq. (2)), the root mean square error (RMSE), chi-squared, and reduced chi squared as computed for the two grain size ranges from **Table 2**, where applicable, for tumbler experiments.  Two experiments are not included in this table; no mass measurements were recorded for P100ig06eq33A and the clasts in P100ig03eq33A were not tumbled. The values in this table are reported with greater precision in the **Supplementary Material** for this article.

Reference Code	*κ*_0,fine_ (km^−1^)	*κ*_1,fine_ (km^−1^)	*κ*_2,fine_	RMSE	*χ*2	*χ*2_ν_	*κ*_0,coarse_ (km^−1^)	*κ*_1,coarse_ (km^−1^)	*κ*_2,coarse_	RMSE	*χ*2	*χ*2_ν_
S253ig02eq33A	n/a	n/a	n/a	n/a	n/a	n/a	4.8E−03	9.7E−04	6.5E−01	9.7E−04	8.8E−02	1.8E−02
S253ig04eq33A	n/a	n/a	n/a	n/a	n/a	n/a	1.3E−08	2.8E−02	2.5E−01	3.3E−03	7.7E−01	1.9E−01
S253ig05eq33A	n/a	n/a	n/a	n/a	n/a	n/a	4.5E−07	2.0E−02	1.7E−01	4.8E−03	2.8E+00	3.1E−01
S253ig03eq33A	n/a	n/a	n/a	n/a	n/a	n/a	1.1E−05	3.0E−03	7.2E−01	1.3E−03	1.4E−01	2.9E−02
S253vc03eq33A	n/a	n/a	n/a	n/a	n/a	n/a	1.3E−08	5.5E−03	6.3E−01	1.1E−03	1.1E−01	2.2E−02
S253co05eq33A	n/a	n/a	n/a	n/a	n/a	n/a	1.3E−07	2.6E−02	8.1E−01	2.5E−03	4.4E−01	1.1E−01
S253me05eq33A	n/a	n/a	n/a	n/a	n/a	n/a	4.8E−05	2.0E−02	7.7E−01	2.3E−03	3.8E−01	9.5E−02
S253ig05eq33B	n/a	n/a	n/a	n/a	n/a	n/a	3.8E−09	4.2E−02	9.5E−01	9.9E−04	4.0E−02	4.0E−02
S253ig05eq33C	n/a	n/a	n/a	n/a	n/a	n/a	4.5E−10	4.9E−02	7.2E−01	8.5E−03	2.9E+00	2.9E+00
P195vc01eq33A	n/a	n/a	n/a	n/a	n/a	n/a	2.2E−09	6.8E−01	5.4E−01	1.4E−02	1.5E+01	3.0E+00
P195vc02eq33A	n/a	n/a	n/a	n/a	n/a	n/a	2.9E−01	4.6E−01	9.0E−01	9.7E−03	1.0E+01	1.3E+00
P195vc06eq33A	n/a	n/a	n/a	n/a	n/a	n/a	5.3E−11	3.5E−01	6.8E−01	1.6E−02	2.9E+01	3.2E+00
P195vc12eq33A	n/a	n/a	n/a	n/a	n/a	n/a	1.8E−02	4.1E−01	3.2E−01	3.5E−03	9.7E−01	1.9E−01
R195vc01eq33A	n/a	n/a	n/a	n/a	n/a	n/a	4.9E−09	5.5E−01	6.3E−01	7.1E−03	2.0E+00	2.0E+00
R195vc02eq33A	n/a	n/a	n/a	n/a	n/a	n/a	8.3E−04	3.7E−01	6.1E−01	3.9E−03	6.1E−01	6.1E−01
R195vc06eq33A	n/a	n/a	n/a	n/a	n/a	n/a	5.7E−05	3.3E−01	3.4E−01	5.3E−03	1.1E+00	1.1E+00
R195vc12eq33A	n/a	n/a	n/a	n/a	n/a	n/a	3.2E−04	3.2E−01	3.6E−01	1.7E−03	1.2E−01	1.2E−01
P100ig06eq33B	8.5E−02	9.5E−02	7.3E−01	7.7E−05	1.6E−03	Inf.	n/a	n/a	n/a	n/a	n/a	n/a
P100ig07nevaA	5.4E−02	1.4E−01	6.5E−01	3.0E−04	3.5E−03	3.5E−03	n/a	n/a	n/a	n/a	n/a	n/a
P100ig06eq33C	4.9E−02	1.2E−01	6.5E−01	4.4E−03	1.7E+00	2.9E−01	n/a	n/a	n/a	n/a	n/a	n/a
P100ig06eq33D	6.5E−02	4.3E−02	8.5E−01	6.9E−03	7.6E+00	5.9E−01	n/a	n/a	n/a	n/a	n/a	n/a
P100ig07nevaB	5.4E−07	1.7E−01	5.5E−01	5.1E−03	1.6E+00	5.2E−01	n/a	n/a	n/a	n/a	n/a	n/a
P100ig04eq33A	2.8E−03	1.6E−01	4.4E−01	3.0E−03	6.5E−01	1.6E−01	n/a	n/a	n/a	n/a	n/a	n/a
P100ig06eq33E	3.0E−06	1.6E−01	3.9E−01	4.8E−03	1.9E+00	3.8E−01	n/a	n/a	n/a	n/a	n/a	n/a
P100gr06eq33A	8.1E−02	4.8E−02	9.5E−01	6.1E−03	2.6E+00	6.5E−01	n/a	n/a	n/a	n/a	n/a	n/a
P100ig04eq45A	2.2E−08	3.3E−01	5.7E−01	1.6E−02	2.0E+01	4.0E+00	n/a	n/a	n/a	n/a	n/a	n/a
P100ig04eq57A	5.5E−08	4.4E−01	4.4E−01	2.2E−02	3.5E+01	8.7E+00	n/a	n/a	n/a	n/a	n/a	n/a
P100ig06eq33F	4.2E−10	1.9E−01	3.9E−01	1.8E−02	2.3E+01	5.7E+00	6.3E−01	1.6E−07	1.6E−03	6.5E−02	3.0E+02	7.5E+01
P100ig06eq33G	2.7E−02	1.7E−01	2.5E−01	2.2E−03	2.5E−01	1.2E−01	1.5E−01	8.5E−03	1.0E+00	2.4E−02	3.0E+01	1.5E+01
P100ig04eq57B	3.9E−06	3.1E−01	2.8E−01	1.7E−02	1.4E+01	6.9E+00	1.6E−04	4.5E−01	4.8E−01	7.2E−03	2.6E+00	1.3E+00
P100ig04eq57C	7.7E−11	3.1E−01	4.4E−01	2.1E−02	3.0E+01	7.5E+00	8.8E−08	4.3E−01	5.0E−01	1.6E−02	1.8E+01	4.5E+00
P100ig06eq33H	1.5E−03	1.5E−01	5.0E−01	4.4E−03	1.3E+00	3.3E−01	3.2E−01	2.0E−02	1.0E+00	1.4E−02	1.3E+01	3.2E+00
P100ig06eq33I	5.9E−02	5.9E−02	7.6E−01	5.4E−03	2.1E+00	5.2E−01	8.5E−02	4.4E−02	1.0E+00	1.0E−02	7.3E+00	1.8E+00
P100ig06eq33J	5.3E−08	1.3E−01	5.2E−01	9.3E−03	6.1E+00	1.5E+00	7.6E−01	1.1E−01	1.0E+00	2.7E−02	5.0E+01	1.3E+01
P100ig03eq33B	1.5E−01	3.2E−06	2.2E−02	7.6E−03	2.3E+00	2.3E+00	8.2E−01	2.0E−06	1.0E−03	9.3E−02	3.5E+02	3.5E+02
P100sg06eq33A	3.9E−11	1.4E−01	5.6E−01	7.4E−03	3.8E+00	9.5E−01	6.1E−01	1.3E−07	1.9E−03	6.2E−02	2.7E+02	6.7E+01
P100ig94nevaA	8.0E−09	8.4E−02	5.1E−01	2.3E−02	9.8E+01	6.5E+00	8.5E−12	4.0E−01	4.5E−01	3.4E−02	2.1E+02	1.4E+01
P100ig55nevaA	3.4E−02	1.8E−01	9.0E−01	1.0E−02	8.0E+00	1.6E+00	3.5E−02	2.2E−01	8.9E−01	9.2E−03	6.7E+00	1.3E+00
P100fc06eq33A	9.8E−07	1.2E−01	4.9E−01	6.3E−03	2.8E+00	6.9E−01	9.7E−05	1.3E−01	5.4E−01	4.6E−03	1.5E+00	3.7E−01
P100fc06eq33B	3.9E−01	2.4E−03	2.7E−03	6.4E−02	2.5E+02	8.2E+01	3.5E−07	5.4E−01	4.7E−01	1.8E−02	1.9E+01	6.5E+00

Two videos are included in the **Supplementary Material** that depict the PVC Titan Tumbler in action. **Video 1** shows the Titan Tumbler spinning in its typical configuration at ∼37 RPM within the walk-in freezer. The other, **Video 2**, shows a down-axis view into the tumbler through a transparent acrylic lid to show six icy clasts and finer fragments rolling and colliding as the barrel spins in slow motion.

## 2 Experimental Design, Materials and Methods

Experiments were devised to investigate the abrasion (i.e., the mechanical processes that cause a particle to lose mass) of water ice clasts under a variety of initial conditions. Different tumblers and methodologies were employed over the course of this work.

### 2.1 Developing icy sediment

Icy clasts were frozen from deionized, degassed water into cubes either 3.3 or 5.7 cm in diameter. Clasts made in this manner were considered to be composed of polycrystalline ice, referred to as “igneous”. Ice clasts were also made from more monocrystalline “sedimentary” ice by grinding “igneous” clasts in a snow-cone machine, sieving to the sand size of interest, packing these grains into cubic molds and saturating with chilled water to be refrozen into new cubes with a dominant ice crystal size.

Clasts made in cubic molds were considered to be equant. Nonequant clasts were made by fragmenting cubic clasts with a hammer to yield angular clasts of various sizes.

### 2.2 Pilot study tumbler

**Fig. 1:**
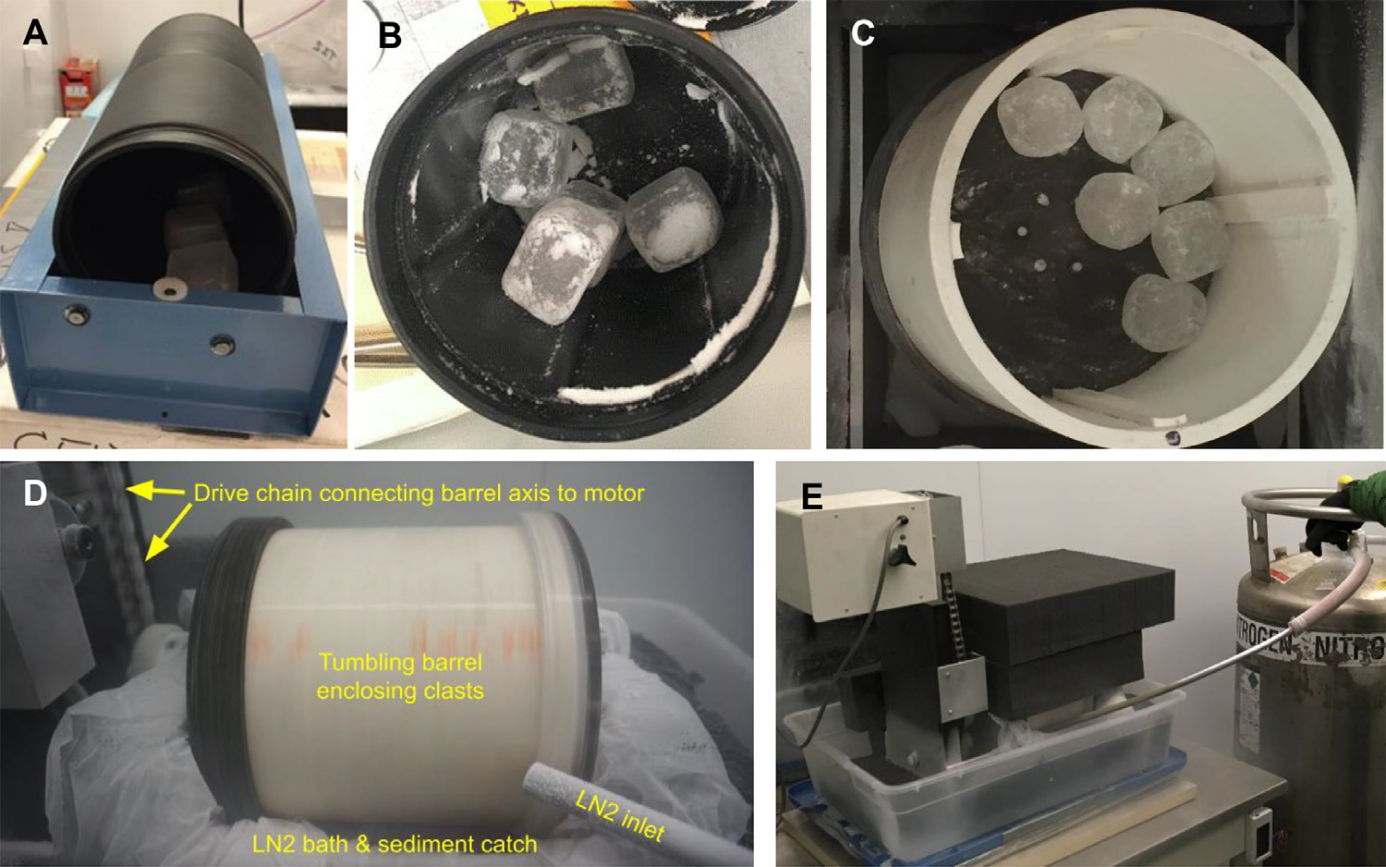
(A) Image of dual-barrel SBR tumbler in chest freezer with end cap removed. (B) View into SBR tumbler with 5 clasts. (C) View into PVC tumbler with 6 clasts. (D) Annotated image from video of PVC Titan Tumbler in action with insulating walls removed. The tumbler spins through a shallow pool of LN_2_ contained by a layer of plastic sheeting, with input from the tube at bottom right. A motor elevated above the apparatus powers the tumbler via the drive chain seen at top left. (E) Wider view of tumbling barrel enclosed in black foam insulation with LN_2_ bath fed by cryogenic storage dewar on right. Modified from Maue et al. [4].

In preliminary experiments, ice clasts were tumbled in an off-the-shelf 11 by 11 cm tumbler composed of dual styrene-butadiene rubber (SBR) barrels with six internal baffles (Fig. 1A and B). These experiments were performed in a chest freezer at ∼253 K. To test the boundary conditions of the system and the planned methodology, varying numbers of clasts and ice types were input to the tumbler.

### 2.3 Improved tumbler design

To run experiments at lower, more Titan-like temperatures, a custom tumbling apparatus was developed. This 15 by 15 cm barrel was built from PVC with four internal baffles. Rotation is driven by a chain connecting the barrel axis to an elevated, adjustable motor (Fig. 1**C–E**). At a rotation speed of 37.5 RPM, 3.3 cm clasts were observed through a transparent acrylic lid to be rolling, rather than slumping or cascading (see **Supplementary Material,** V**ideos**).

Tuning experiments with varying number of clasts were performed at ∼195 K, cooled by packing dry ice around the exterior of the barrel. Experiments were also performed with and without the PVC barrel's interior being coated in rubber to detect a possible effect of abrasion due to the barrel material.

For Titan-like experiments at ∼100 K, the barrel rotated in a shallow bath of liquid nitrogen (LN_2_). A thermocouple wired through the barrel's axis monitored the internal temperature so that LN_2_ levels could be adjusted to maintain temperatures at or lower than ∼100 K. The remaining 25 of 41 tumbling experiments were performed at this temperature. Prior to tumbling, the barrel was cooled with an internal and external wash of LN_2_.

Before tumbling ice clasts in the LN_2_-cooled barrel, clasts initially frozen at 253 K were first lowered down to LN_2_ temperatures. This tempering was performed by placing clasts in a lightly insulated bin and lowering that bin into an LN_2_ bath for ten minutes. Tempering showed signs of changing the internal structure of clasts (see Fig. S10 in Maue et al. [4]). Two experiments (P100ig94nevaA and P100ig55nevaA) involved disaggregating tempered clasts into nonequant shapes before tumbling to expedite the initial fragmentation.

### 2.4 Sieving, weighing, and imaging

For the lower temperature experiments, procedures were modified to suit the ice behavior. At 253 and 195 K, clasts experienced no fragmentation and mostly abraded via attrition such that measurements could follow the same clasts that were initially input. At ∼100 K, clasts experienced increased fragmentation so that tracking mass and roundness required focusing on sieved grain size ranges.

Between tumbling, measurements usually took 15 and 20 min to complete. Clasts tumbled in experiments at 253 and 195 K were weighed and imaged between tumbling intervals in a 253 K chest freezer. Experiments at ∼100 K had the entire tumbler apparatus placed inside a 253 K walk-in freezer, where measurements were also taken. For experiments where the grain size distribution was measured, clasts were transferred to the sieves and fines were brushed from the barrel interior and off the largest clasts into the sieves. Sieve sizes ranging from 63 µm to 16 mm were used, but most experiments involved sieving for grain sizes <0.125, 0.125–1, 1–4 4–8, 8–16, and >16 mm.

At 253 and 195 K, the initial clasts remained largely intact and so the same set were weighed at each interval, staying larger than 16 mm for the duration of the experiment. These clasts were weighed on a tared scale then returned to the barrel for continued tumbling as any fines generated were removed from the experiment.

At ∼100 K, clasts fragmented into a broad grain size range such that a size cutoff was selected to determine which grains would be returned to the barrel. In early LN_2_ experiments with no sieving, the largest clasts (approximately coarser than pebble-scale as defined by Wentworth [5]) were isolated to be weighed on a tared scale. In later LN_2_ experiments that incorporated sieving, the size threshold was usually 1 mm and all clasts were weighed in their respective sieves before subtracting the weight of the sieve to get the true mass of that grain size range. Grains finer than the threshold size were removed from the experiment whereas coarser grains were set aside for imaging or returned to the barrel.

After weighing, the largest clasts were transferred to a black felt mat for imaging. Imaging was done for clasts >16, 8–16, and 4–8 mm, if present and preserved. Images were taken at a standard distance using an iPhone camera. Clasts >16 mm were rotated to be imaged on three sides. After imaging, these coarse grains, and any unimaged grains coarser than the selected size threshold were returned to the barrel for the next tumbling interval.

### 2.5 Estimating transport distance

The estimated transport distance of tumbled sediment is computed as the product of rolling speed and time. For the 15 cm-diameter (47 cm-circumference) PVC barrel spinning at 37 RPM, a circular velocity of 0.29 m/s is used to convert time spent tumbling into transport distance. Variable speeds in experiments with the 11 cm-diameter SBR barrel were calculated in the same manner. For the larger PVC barrel, the frequently used tumbling intervals of 15 min and 1 h correspond to estimated transport distances of 0.27 and 1.08 km, respectively.

### 2.6 Best-fit abrasion rates

The mass measurements from each experiment were fit by models used in previous tumbler studies. The classic exponential fining model of Sternberg [2], arranged for mass, follows

(1) $$M=M_{0}e^{-kx},$$

where *x* is distance (km), *k* is the mass-loss coefficient (km^−1^), and *M*_0_ and *M* are the mass initially and at distance *x*, respectively. A more recent model by Mikoš [3] may more accurately describe the mechanisms of fluvial abrasion with

(2) $$M=M_{0}e^{-\left[\kappa_{0}+\kappa_{1}{\left(x+x_{0}\right)}^{-\kappa_{2}}\right]x},$$

where *κ*_0_ is accounts for mass loss due to attrition (km^−1^; similar to Sternberg's *k*), *κ*_1_ accounts for mass loss due to fragmentation (km^−1^), *κ*_2_ is an index of the rate at which fragmentation's role diminishes, and *x*_0_ is the initial distance (km)—which is equated to zero in application to these experiments. The processes referred to here as attrition and fragmentation, Mikoš [3] refers to as abrasion and chipping, respectively.

Warmer experiments without fragmentation could apply these models to the original set of clasts and their mass-loss over distance. For colder LN_2_ experiments with enhanced fragmentation, the models were applied to the mass-loss of grains coarser than a defined “fine” size (e.g., 1 mm) and “coarse” size (e.g., 16 mm) reported in Table 2 so that Tables 2 and 3 report *k*_fine_, *k*_coarse_, *κ*_0,fine_, *κ*_0,coarse_, and etc.

### 2.7 Image processing to quantify shape

Changes in shape were investigated with a roundness index,

(3) $$R=\frac{4\pi A}{P^{2}},$$

where *A* is area and *P* is perimeter as viewed in cross section. This index approaches one as the shape of the clast in plan view approaches that of a circle. Mean, median, and standard deviation is reported for clasts of a given grain size range, incorporating three views for clasts >16 mm.

The MATLAB script used for computing roundness indices from images of clasts is included in the **Supplementary Material**. This script is borrowed from the Supplementary Material of Manga et al. [1], with minor changes in order to save the output image and generate an output table of the computed roundness indices.

To accurately quantify a roundness index for each clast, three variables can be adjusted in the code: *s_wavelength, threshold*, and *min_pixels*. The *s_wavelength* is set to use every Nth pixel for the perimeter calculation to smooth out artifacts and in these experiments was set equal to 3 or 4. The *threshold* sets the brightness value that defines the edge of bright clasts versus the dark background and was generally ∼0.4 in these experiments but could range from roughly 0.2–0.6. The *min_pixels* parameter is set to remove all objects containing fewer pixels than its value, for which 500 was used.

For a given image, choosing the right *threshold* is important for accurately delineating the edge of each clast. This value was adjusted until boundaries appeared correct so that background ice dust is not counted as part of an ice clast and translucent sections of ice are not counted as background. As image outputs are inspected in this qualitative manner, it is possible that a range of parameters can produce visually similar outputs while computing slightly different roundness indices. If no *threshold* value seems to appropriately define the boundaries of a clast, as occurred in a few rare cases, images were digitally edited to remove erroneous background features or separate overlapping clasts. More details on the influence of these parameters and other sources of error in determining roundness indices can be found in the Supplementary Material of Maue et al. [4].

## Ethics Statement

No human or animal subjects were involved in data collection.

## CRediT authorship contribution statement

**Anthony D. Maue:** Data curation, Formal analysis, Investigation, Methodology, Writing – original draft, Writing – review & editing, Validation, Visualization. **Joseph S. Levy:** Conceptualization, Funding acquisition, Investigation, Methodology, Project administration, Writing – review & editing. **Devon M. Burr:** Conceptualization, Funding acquisition, Investigation, Methodology, Writing – review & editing. **Patrick R. Matulka:** Formal analysis, Investigation, Methodology. **Erica Nathan:** Formal analysis, Investigation, Methodology, Writing – review & editing.

## Declaration of Competing Interest

The authors declare that they have no known competing financial interests or personal relationships which have, or could be perceived to have, influenced the work reported in this article.
